# Associations of health-related quality of life with depression and stigma in MERS-CoV survivors during the recovery period

**DOI:** 10.1097/MD.0000000000029440

**Published:** 2022-06-24

**Authors:** So-Hyun Ahn, Jeong Lan Kim, So Hee Lee, Hye Yoon Park, Jung Jae Lee, Haewoo Lee

**Affiliations:** a Department of Psychiatry, Chungnam National University Hospital, Daejeon, Republic of Korea; b Department of Psychiatry, National Medical Center, Seoul, Republic of Korea; c Department of Psychiatry, Seoul National University Hospital, Seoul, Republic of Korea; d Department of Psychiatry, Dankook University School of Medicine, Cheonan, Chungnam, Republic of Korea; e Department of Psychiatry, Seoul Medical Center, Seoul, Republic of Korea.

**Keywords:** depression, mental health, Middle East respiratory syndrome, quality of life, stigma

## Abstract

We explored factors related to health-related quality of life (HRQOL), including psychiatric symptoms and stigma related to Middle East respiratory syndrome coronavirus (MERS-CoV) infection, among MERS-CoV survivors during the recovery period.

Sixty-three MERS-COV survivors were recruited from five hospitals for a cohort study, one year after their infection in 2015. The subjects’ demographic information and medical conditions associated with MERS-CoV were recorded. HRQOL was evaluated using the Short Form-8 Health Survey (SF-8). Depression, post-traumatic stress symptoms, chronic fatigue, and perceived stigma were assessed using several questionnaires

The mean physical component summary (PCS) and mean mental component summary (MCS) of the SF-8 score were below 50 T (43.47 ± 9.60, 45.74 ± 10.18). Depression, chronic fatigue, posttraumatic stress symptoms and stigma were negatively correlated with the SF-8 PCS and MCS. Multivariate logistic regression analysis showed that the PCS was associated with stigma (OR 8.66, 95% CI 1.96–38.23), whereas MCS was associated with depression (OR 26.62, 95% CI 3.56–198.85).

The estimated HRQOL of MERS-CoV survivors during recovery was poor and appeared to be associated with depression and MERS-related stigma.

## 1 Introduction

Middle East respiratory syndrome coronavirus (MERS-CoV), an acute respiratory infection caused by a coronavirus, was first reported in Saudi Arabia in September 2012.^[1]^ South Korea experienced an outbreak of MERS-CoV, that began on May 20, 2015, when a man who returned from a trip to Saudi Arabia was confirmed to have MERS-CoV. The MERS-CoV epidemic lasted 217 days. Of 186 patients confirmed to have MERS-CoV, 38 died, with a mortality rate of 20.4%.^[2]^

Outbreaks of an infectious viruses tend to cause a variety of health and social problems. Even before the MERS-CoV epidemic, new infectious diseases such as severe acute respiratory syndrome (SARS), Ebola virus disease (EVD), and influenza A caused deaths, as well as high social and psychological costs.^[3]^ Another new infectious disease, coronavirus disease 2019 (COVID-19), is currently a pandemic, resulting in a global public health crisis around the world. Evidence from previous large-scale health outbreaks suggests that this type of event has a tremendous impact on mental health, quality of life, and physical health.^[4]^ SARS patients were found to experience greater negative psychological distress than the general population,^[5]^ and psychological stress persisted in these patients for 1 year after recovery.^[6]^ A study that monitored patients with SARS for 46 months after discharge from the hospital found that 44.1% developed post-traumatic stress disorder (PTSD) during the follow-up period and had a lower quality of life.^[7]^

A retrospective chart review of isolated inpatients with MERS-CoV at a hospital in Korea reported that 70.8% of them had psychiatric symptoms, and 41.7% were diagnosed with mental illness and prescribed medication during hospitalization.^[8]^ More than a third of MERS-CoV survivors suffer from post-traumatic symptoms, sleep problems, anxiety, and depression for 1 year after infection.^[9]^ One year after MERS-CoV infection, depression persisted in association with chronic fatigue, which indirectly affected post-traumatic symptoms (PTS).^[10]^ Those who reported chronic fatigue syndrome at 1 year after MERS-CoV infection were more likely to experience suicidality during the 2-year follow-up than those who did not.^[11]^

Studies on the quality of life of patients with new infectious diseases have mostly reported associations with physical condition or lung function.^[12,13]^ However, considering that psychiatric symptoms affect the quality of life of patients with other infectious diseases,^[14]^ it is reasonable to hypothesize that not only physical symptoms, but also mental health status, would affect the quality of life of patients with new infectious diseases. Previous studies on SARS and EVD survivors demonstrated that health-related quality of life (HRQOL) was associated with five key domains: physical functioning, psychological functioning, social support, social functioning (SF), and demographic factors.^[15]^ Recent studies on HRQOL dimensions in COVID-19 survivors suggested that physical symptoms, anxiety, trauma, economic loss, place-based identity, self-stigma, health self-interventions, and changing lifestyles were affected in an intertwined manner.^[16]^

Few follow-up studies on the HRQOL and mental health of MERS-CoV survivors have been reported. Thus, we conducted a cross-sectional cohort study of MERS-CoV survivors after one year to evaluate the associations between HRQOL and depression, PTS symptoms, chronic fatigue, and MERS-CoV-related stigma. Our hypothesis was that HRQOL among MERS-CoV survivors would be related to medical and psychological difficulties, as well as psychosocial stigma.

## 2 Methods

### 2.1 Participants

The five centers that treated most South Korean patients during the MERS-CoV outbreak constructed cohorts of patients monitored during the recovery period. All survivors who were confirmed as having MERS-CoV infection were asked to participate in the present study, which was conducted 12 months after the MERS-CoV infection. Participants was requested through mail and telephone calls.

Of 148 survivors, 72 (48.65%) agreed to participate and were assessed 12 months after the MERS-CoV outbreak. The patients visited the hospital for regular check-ups and completed a questionnaire on their health status. A total of 63 patients completed the questionnaire, including 15 from the National Medical Center, 17 from Seoul National University Hospital, 13 from Chungnam National University Hospital, 12 from Seoul Medical Center, and 6 from Dankook University Hospital.

### 2.2 Assessments

Data on demographic variables including sex, age, education, marital status, and employment were collected, along with medical illnesses prior to MERS-CoV and history of visits to the psychiatric department. Data on patients’ medical conditions, including pneumonia, ventilator care, and extracorporeal membrane oxygenation, were also gathered.

The patients completed the following self-reported questionnaires 12 months after MERS-CoV infection: Patient Health Questionnaire-9 (PHQ-9), Impact Event Scale-Revised (IES-R), Fatigue Severity Scale (FSS), MERS stigma scale, and Short Form-8 Health Survey (SF-8).

#### 2.2.1 Patient health questionnaire-9

The PHQ-9 is widely used to assess self-reporting depression. The PHQ was developed in 1999 by Spitzer and Kroenke based on the Diagnostic and Statistical Manual of Mental Disorders, Fourth Edition criteria for depression.^[17]^ It consists of nine questions scored from 0 to 3 according to the frequency of the symptoms. The total score ranges from 0 to 27 points; scores of 0 to 4, 5 to 9, 10 to 14, 15 to 19, and 20 to 27 indicate no, mild, moderate, moderately severe, and severe depression, respectively].^[18]^ The Korean version of the PHQ-9 was validated by Choi et al,^[19]^ and the reliability of the entire questionnaire was 0.93. In the present study, patients with a total score of 10 or higher were classified as having depression.

#### 2.2.2 Impact event scale-revised

The IES was used to measure PTSD symptoms associated with exposure to traumatic events (MERS-CoV infection in our study). The IES was developed by Horowitz et al in 1979, and revised by Weiss and Marmar in 1997.^[20,21]^ The IES consists of 22 questions spread over three subscales: intrusion, avoidance, and hyperarousal. Each question is scored from 0 to 4. PTSD was diagnosed when the IES-R score was > 25 points (sensitivity, 1.00; specificity, 0.51). In this study, we used the IES-R, which was translated into Korean by Eun et al and has proven reliability and validity. The reliability of the questionnaire was 0.97.^[22]^ In this study, patients with a total score of ≥ 25 were classified as having PTS symptoms.

#### 2.2.3 Fatigue severity scale

The FSS is a fatigue scale developed in 1989 by Krupp et al,^[23]^ that provides a measure of fatigue severity and functionality over the past week and comprises nine items, each of which is assigned 1 to 7 points. The final FSS score is the average value divided by nine after the scores for each question have been combined; higher scores indicate greater fatigue. In a study of South Koreans, the optimum cutoff point for the FSS for distinguishing between fatigue and control groups was 3.22, with 84.1% sensitivity and 85.7% specificity.^[24]^ This cut-off point was also used in this study.

#### 2.2.4 Assessment of perception and experience of MERS-CoV (MERS stigma scale)

MERS-related stigma was assessed using a modified version of the 40-item Berger Human Immunodeficiency Virus (HIV) Stigma Scale and the 8-item short version of the HIV stigma scale.^[25,26]^ The Berger scale measures four dimensions of HIV stigma: personalized stigma, that is, perceived stigmatizing consequences of others knowing about one's HIV status; disclosure concerns, that is, fear of disclosing one's own HIV status as that those who know would tell others; negative self-image, that is, experiencing oneself as tainted and not as good as others because of HIV status; and concerns with public attitudes, that is, preconceptions of what people might think about a person with HIV. The MERS stigma scale uses a 4-point Likert-type scale ranging from 0 to 3 (“strongly disagree” to “strongly agree”), where higher scores indicate higher levels of stigma.^[27]^ Thus, the overall score ranges from 0 to 24. The participants in this study were divided into two groups via median split, according to the scores on each subscale and the total score.

#### 2.2.5 SF-8 (HRQOL)

HRQOL was assessed using the SF-8.^[28]^ This scale comprises eight items/sub-dimensions: general health, physical function, role-physical health (RP), bodily pain, vitality, SF, mental health, and role-emotional health (RE). Each item is scored on a 5- or 6-point Likert-type scale. The items were converted into a 0 to 100 scale using the T-score (mean = 50, SD = 10). Two summary scores, the physical component summary (PCS) and mental component summary (MCS), were also calculated from the weighted sums of the relevant subscale scores. Higher PCS or MCS scores indicate better physical or mental quality of life. The Korean version of SF-8 has been validated.^[29]^

### 2.3 Statistical analyses

Participants’ demographic characteristics and medical conditions were evaluated as categorical variables and presented as proportions. Independent t-tests were used to compare the characteristics according to the SF-8 PCS and MCS. To evaluate the relationships between SF-8 and psychiatric symptom scale scores, we used Pearson's correlation analysis. To determine the factors affecting HRQOL PCS and MCS, we used binary logistic regression analysis. We classified subjects with a T-score of <40 as having poor HRQOL based on the SF-8 PCS and MCS scores. Participants were classified into two groups based on the median MERS stigma scale score. The SF-8 PCS and MCS constituted the outcome values. Depression, PTS symptoms, chronic fatigue, and MERS-related stigma were entered separately into the regression model. Then, variables with *P*-values < .1 were entered simultaneously. Statistical significance was set at *P* < .05. All statistical analyses were performed using the SPSS software (version 24.0; SPSS Inc., Chicago, IL, USA).

## 3 Results

A total of 63 patients (39 (61.9%) men and 24 (38.1%) women) with an average age of 49.21 ± 12.57 years (range: 24–80 years) were included. Twenty-one (73.4%) patients had pneumonia, 12 (19%) were treated using a ventilator, and four (6.3%) required extracorporeal membrane oxygenation. Patients who had previously visited a psychiatrist (n = 10, 15.9%) had lower scores on the mental component of HRQOL (t = 2.476, *P* = 0.032). There were no significant differences in SF-8 scores according to demographic characteristics or medical conditions (Table 1).

**Table 1:** General characteristics and medical conditions of patients (N = 63).

		SF-8PCS			SF-8MCS		
Variables	N(%)	Mean(SD)	*t*	*P*	Mean(SD)	*t*	*P*
Sex							
Male	39 (61.9)	44.74 (8.59)	1.459	.150	46.97 (10.18)	1.230	.223
Female	24 (38.1)	41.14 (10.87)			43.74 (10.07)		
Age (years)							
<50	34 (54.0)	45.13 (9.74)	1.600	.115	46.41 (10.05)	0.563	.576
≥50	29 (46.0)	41.30 (9.17)			44.95 (10.45)		
Education							
≤12 years	30 (47.6)	42.01 (8.86)	1.069	.289	46.03 (9.59)	−.214	.832
>13 years	33 (52.4)	44.60 (10.20)			45.48 (10.83)		
Marital status							
Married	50 (79.4)	43.77 (8.60)	0.503	.623	46.03 (9.94)	0.437	.664
Unmarried/divorced/separated	13 (20.6)	41.84 (13.08)			44.63 (11.41)		
Employment							
Health care provider	18 (28.6)	43.95 (9.98)	−.303	.763	48.16 (8.11)	−1.198	.235
Non-health care provider	45 (71.4)	43.13 (9.55)			44.77 (10.83)		
Psychiatry visit history							
Yes	10 (15.9)	40.31 (9.02)	1.099	.276	47.47 (8.55)	2.476	.032^∗^
No	53 (84.1)	43.94 (9.68)			36.56 (13.44)		
Pneumonia							
Yes	21 (33.3)	42.52 (9.52)	0.493	.624	43.79 (11.80)	1.077	.286
No	42 (66.7)	43.79 (9.73)			46.72 (9.27)		
Ventilator care							
Yes	12 (19.0)	43.12 (9.82)	0.099	.922	41.92 (11.57)	1.459	.150
No	51 (81.0)	43.43 (9.65)			46.64 (9.74)		
ECMO							
Yes	4 (6.3)	43.21 (14.35)	0.034	.973	45.52 (12.08)	0.045	.964
No	59 (93.7)	43.38 (9.37)			45.76 (10.16)		
Underlying disease							
Yes	20 (31.7)	40.61 (8.99)	1.571	.121	45.18 (10.52)	0.294	.770
No	43 (68.3)	44.65 (9.70)			46.00 (10.14)		

ECMO = extracorporeal membrane oxygenation, MCS = mental component summary, PCS = physical component summary, SD = standard deviation, SF-8 = Short Form-8 Health Survey.

∗ *P* < .05

The mean PCS score was 43.47 ± 9.60, and the mean MCS score was 45.74 ± 10.18. The mean scores on the eight subdimensions of the SF-8 were also lower than 50 T. Higher scores on the PHQ-9, FSS, IES-R, and MERS stigma scale correlated with lower scores SF-8 PCS scores (*r* = –0.505, *P* = .000; *r* = –0.513, *P* = .000; *r* = –0.424, *P* = .000; *r* = –0.365, *P* = .003, respectively). Higher scores on the PHQ-9, FSS, IES-R, and MERS stigma scale also correlated with lower scores on the SF-8 MCS (*r* = –0.741, *P* = .000; *r* = –0.645, *P* = .000; *r* = –0.658, *P* = .000; *r* = –0.265, *P* = 0.036, respectively). The PHQ-9, FSS, and IES-R scores were negatively correlated with the scores on the eight subdimensions of the SF-8 (all *P* = .000). The MERS stigma scale score negatively correlated with physical functioning, RP, bodily pain, SF, and RE (Table 2).

**Table 2:** Correlations of SF-8 dimensions with other mental health (N = 63).

SF-8 dimensions	Mean (SD)	PHQ-9	*P*	FSS	*P*	IES-R	*P*	Perceived Stigma	*P*
1. GH	40.29 (6.54)	−0.580^∗∗∗^	.000	−0.512^∗∗∗^	.000	−0.538^∗∗∗^	.000	−0.218	.086
2. PF	44.66 (8.17)	−0.513^∗∗∗^	.000	−0.515^∗∗∗^	.000	−0.439^∗∗∗^	.000	−0.349^∗∗^	.005
3. RP	45.29 (8.81)	−0.558^∗∗∗^	.000	−0.551^∗∗∗^	.000	−0.444^∗∗∗^	.000	−0.353^∗∗^	.005
4. BP	47.20 (9.25)	−0.534^∗∗∗^	.000	−0.470^∗∗∗^	.000	−0.469^∗∗∗^	.000	−0.363^∗∗^	.004
5.VT	43.66 (8.21)	−0.594^∗∗∗^	.000	−0.529^∗∗∗^	.000	−0.452^∗∗∗^	.000	−0.203	.110
6. SF	46.95 (8.68)	−0.625^∗∗∗^	.000	−0.691^∗∗∗^	.000	−0.544^∗∗∗^	.000	−0.309^∗^	.014
7. MH	47.77 (9.76)	−0.712^∗∗∗^	.000	−0.562^∗∗∗^	.000	−0.609^∗∗∗^	.000	−0.226	.075
8. RE	42.97 (7.86)	−0.593^∗∗∗^	.000	−0.622^∗∗∗^	.000	−0.604^∗∗∗^	.000	−0.409^∗∗^	.001
9. PCS	43.37 (9.60)	−0.505^∗∗∗^	.000	−0.513^∗∗∗^	.000	−0.424^∗∗∗^	.000	−0.365^∗∗^	.003
10. MCS	45.74 (10.18)	−0.741^∗∗∗^	.000	−0.645^∗∗∗^	.000	−0.658^∗∗∗^	.000	−0.265^∗^	.036

BP = bodily pain, FSS = Fatigue Severity Scale, GH = general health, IES-R = Impact Event Scale-Revised, MCS = mental component summary, MH = mental health, PCS = physical component summary, PF = physical function, PHQ-9 = Patient Health Questionnaire-9, RE = role-emotional, RP = role-physical, SD = standard deviation, SF = social functioning, SF-8 = Short Form-8 Health Survey, VT = vitality

∗ *P* < .05

∗∗ *P* < .01

∗∗∗ *P* < .001

To analyze the factors affecting HRQOL, we divided the subjects into two groups based on PCS and MCS cut-off scores of 40. There were 22 (34.9%) participants subjects in the poor PCS group, and 13 (20.6%) in the poor MCS group. In univariate analysis, depression (odd ratio [OR], 5.83; 95% CI, 1.75–19.44), chronic fatigue (OR, 8.22; 95% CI, 2.47–27.36), PTS symptoms (OR, 5.18; 95% CI, 1.69–15.89), and stigma (OR, 12.27; 95% CI, 3.40–44.36) were significantly associated with PCS. Depression (OR, 40.33; 95% CI, 7.14–227.81), chronic fatigue (OR, 5.44; 95% CI, 1.33–22.30), and PTS symptoms (OR, 11.69; 95% CI, 2.31–49.00) were significantly associated with MCS. In the multivariate analysis, stigma was associated with poorer quality of life in terms of PCS (OR 8.66, 95% CI 1.96–38.23). In contrast, depression was associated with poorer quality of life in terms of MCS (OR 26.62, 95% CI 3.56-198.85) (Tables 3 and 4).

**Table 3:** Factor associated with PCS of SF-8.

	Total	PCS < 40 T					
Variable	N(%)	Unadjusted OR	95% CI	*P*-value	Adjusted OR^∗^	95% CI	*P*-value
Depression				.003^∗∗^			.176
PHQ-9 score < 10	46	1			1		
PHQ-9 score ≥ 10	17	5.83	1.75–19.44		3.20	0.59–17.22	
Fatigue				.000^∗∗∗^			.076
FSS < 3.22	34	1			1		
FSS ≥ 3.22	29	8.22	2.47–27.36		4.07	0.86–19.23	
PTS symptoms				.003^∗∗^			.607
IES-R < 25	36	1			1		
IES-R ≥ 25	27	5.18	1.69–15.89		0.62	0.10–3.87	
Perceived stigma				.000^∗∗∗^			.004^∗∗^
Under median	34	1			1		
Under median	29	12.27	3.40–44.36		8.66	1.96–38.23	

FSS = Fatigue Severity Scale, IES-R = Impact Event Scale-Revised, OR = odd ratio, PCS = physical component summary, PHQ-9 = Patient Health Questionnaire-9, PTS = Post-traumatic stress, SF-8 = Short Form-8 Health Survey

∗ Adjusted for depression, fatigue, PTS, and stigma.

∗∗ *P* < .01

∗∗∗ *P* < .001

**Table 4:** Factor associated with MCS of SF-8.

	Total	MCS < 40 T					
Variable	N(%)	Unadjusted OR	95% CI	*P*	Adjusted OR^∗^	95% CI	*P*
Psychiatry visit history				.194			
No	53	1					
Yes	10	3.26	0.76–13.95				
Depression				.000^∗∗∗^			.001^∗∗^
PHQ-9 score < 10	46	1			1		
PHQ-9 score ≥10	17	40.33	7.14–227.81		26.62	3.56–198.85	
Fatigue				.026^∗∗^			.803
FSS <3.22	34	1			1		
FSS ≥3.22	29	5.44	1.33–22.30		0.73	0.06–8.47	
PTS symptoms				.001^∗∗^			.401
IES-R <25	36	1			1		
IES-R ≥25	27	11.69	2.31–59.00		3.00	0.23–39.10	
Perceived stigma				.071			.939
Under median	34	1			1		
Under median	29	3.38	0.91–12.47		1.08	0.15–8.08	

FSS =  = Fatigue Severity Scale, IES-R = Impact Event Scale-Revised, MCS = mental component summary, OR = odd ratio, PHQ-9 = Patient Health Questionnaire-9, PTS = Post-traumatic stress, SF-8 = Short Form-8 Health Survey

∗ Adjusted for depression, fatigue, PTS, and stigma.

∗∗ *P* < .01

∗∗∗ *P* < .001

## 4 Discussion

We followed up patients with MERS-CoV 12 months after the infection to evaluate the association between HRQOL and mental health. The HRQOL of MERS-CoV patients during the recovery period was generally poor, and correlated with depression, chronic fatigue, post-traumatic symptoms (PTS), and stigma. Stigma was associated with the physical component of HRQOL, whereas depression was associated with the mental component of HRQOL.

Our findings are similar to those reported for the quality of life of other infectious disease survivors. In a recent cross-sectional analysis of hospitalized COVID-19 survivors at 2 months after discharge, the quality of life measured by the SF-12 was 42.5 ± 11.2 for PCS and 45.5 ± 11.5 for MCS, and approximately 40% of participants were determined to have a poor quality of life.^[30]^ A study analyzing 117 SARS survivors showed that the overall quality of life, as measured by the 36-Item Short-Form Health Survey (SF-36), decreased at three months and had not returned to normal at one year.^[31]^ In a study of survivors of avian influenza A virus infection in China, the RP and RE scores on the SF-36 were lower than those of the general population.^[32]^ The quality of life of SARS survivors after 30 months was generally lower than that of Hong Kong's general population in all eight domains measured using the SF-36.^[33]^

Our finding that depression affects the mental component of HRQOL was not surprising. Massive infectious disease outbreaks can be regarded as mental health catastrophes, with an impact that persists during recovery.^[33]^ Depression was negatively correlated with quality of life in our subjects, and the group with depression was significantly more likely to have low scores for the mental component of quality of life than the group without depression.^[34]^ Presumably, this was due to the negative effects of depression on daily functioning and other related domains. The results of an online survey of clinically stable COVID-19 patients conducted during isolation in hospital, showed that 43.1% of the participants exhibited depression, and patients with depression had a lower quality of life than those without.^[35]^ A study from Singapore, which evaluated mental functioning and quality of life in 63 SARS survivors at 3 months after discharge, showed that quality of life after 3 months was poor and was correlated with depression, anxiety, and PTS symptoms.^[36]^

In the present study, stigma related to MERS-CoV infection was associated with quality of life independent of other mental health issues. The group thar perceived stigma was significantly more likely to have poor physical quality of life than the group that did not perceive stigma. Stigmatization often contributes to poor global health outcomes, particularly the stigma related to the diagnosis and treatment of infectious diseases and mental illness.^[37]^ The stigma associated with HIV/AIDS and EVD is associated with isolation, bullying, physical violence, and poor quality of life. The stigma associated with HIV/AIDS and EVD is associated with isolation and bullying, physical violence, and a poor quality of life.^[35]^ Exposure to infectious diseases leads to interpersonal and psychological avoidance, to prevent the disease from spreading; the general public tends to be afraid of, and stigmatizes, those exposed.^[38,39]^ Uncertainties in physical status may lead patients to self-stigmatize.^[16]^ In South Korea, the infection rate of MERS-CoV during the outbreak was higher than in other countries, which caused people to be more fearful, and the stigma associated with infection was greater.^[40]^ At the time of acute infections, including MERS-CoV infection, the unsettling and initially obscure clinical and pathological characteristics, and the associated quarantine procedures, may result in disproportionate and unhelpful labeling of patients.

Research on the relationship between stigma and quality of life among people living with HIV has emphasized the need for interventions designed to reduce perceived stigma and strengthen social support from families.^[14]^ A study on stigma and quality of life in COVID-19 survivors suggested that online treatments, such as cognitive behavioral therapy, could be a useful option in the context of a pandemic.^[30]^

Our study has several limitations. First, only 48% of the survivors were enrolled. Second, we did not standardize the stigma scale using translation and reverse translation. However, there is no gold standard for quantitative measurement of stigma. Various instruments are available, but these mainly address HIV/AIDS, tuberculosis, mental health, cancer, epilepsy, asthma, and obesity.^[40]^ The Berger HIV stigma scale mentioned above best matcheed the conceptual framework of our study. Finally, as this was a cross-sectional study of patients at 12 months post-infection, it was difficult to determine causality in the relationships between quality of life and other variables.

Despite these limitations, this study is relevant because there has been little research on the quality of life, mental health, or stigmatization of patients with infectious diseases, especially in Korea. Face-to-face interviews with psychiatrists represent the main strength of this research.

## 5 Conclusion

In conclusion, the quality of life of MERS-CoV survivors during recovery appears to be poor, and this seems to be related to depression and stigma.

## Author contributions

**Conceptualization:** Jeong Lan Kim, So Hee Lee

**Formal analysis:** So-Hyun Ahn

**Funding acquisition:** So Hee Lee

**Investigation:** Jeong Lan Kim, So Hee Lee, Hye Yoon Park, Jung Jae Lee, Haewoo Lee

**Methodology:** So-Hyun Ahn, Hye Yoon Park, Jung Jae Lee, Haewoo Lee

**Supervision:** Jeong Lan Kim

**Writing – original draft:** So-Hyun Ahn

**Writing – review & editing:** Jeong Lan Kim, So Hee Lee, Hye Yoon Park, Jung Jae Lee, Haewoo Lee
